# Effects of anthropometry, age and sex on center of pressure displacement evoked by small trunk perturbations in balance tests

**DOI:** 10.1007/s00421-026-06210-z

**Published:** 2026-04-15

**Authors:** Maria Paterna, Francesca Alba, Emiliano Bottoni, Carlo De Benedictis, Daniela Maffiodo, Walter Franco, Zeevi Dvir, Carlo Ferraresi, Silvestro Roatta

**Affiliations:** 1 Department of Mechanical and Aerospace Engineering, Politecnico di Torino, Turin, Italy; 2 Department of Physical Therapy, The Gray Faculty of Medical and Health Sciences, Tel Aviv University, Tel Aviv, Israel; 3 Department of Neuroscience, University of Torino, Turin, Italy

**Keywords:** Postural reaction, Center of pressure, Balance control, Human balance modeling, Stability, Posturography

## Abstract

**Purpose:**

Balance control can be evaluated by studying the displacement of the center of pressure (CoP) during dynamic posturographic trials. However, differences in anthropometric characteristics may affect the measurement and increase intersubject variability, possibly leading to misinterpretation. This study evaluates how sex, age, and anthropometric characteristics (weight, height, and foot length) influence the CoP displacement to postural perturbations.

**Methods:**

Seventy-two healthy subjects, 31 women and 41 men, were subjected to five small impulsive perturbations applied to their back. Balance performance was quantified by three CoP parameters: the ratio between the maximum CoP displacement and the impulse of the perturbation (Δ*CoP*_*n*_), the latency (*Lat*), and the duration (*Dur*) of the CoP response.

**Results:**

A linear regression analysis evidenced that body weight and height exhibited a negative and positive relation with Δ*CoP*_*n*_, respectively, which explained 48% of the Δ*CoP*_*n*_ variability. These relations were confirmed by simulations with a single-link biomechanical model. On the other hand, the latency and the duration of the CoP response were affected neither by the anthropometric characteristics nor by sex.

**Conclusion:**

Findings from the present study have an impact on test protocols, especially when comparing data collected from heterogeneous populations, in which case normalization with respect to the subjects’ height and weight may be necessary to use the Δ*CoP*_*n*_ as a postural performance index.

## Introduction

One of the most basic human abilities is balance. It is essential for carrying out daily tasks as well as for managing unpredictable perturbations resulting from interaction with the environment. This ability may be compromised by disorders affecting central mechanisms (such as stroke (Chen et al. 2024), neurodegenerative diseases (Sarasso et al. 2023), cerebellar degenerations (McNames et al. 2025), and demyelinating diseases (Corrini et al. 2023)) or the peripheral nervous system (e. g., vestibular disfunctions (Donovan et al. 2023), peripheral neuropathies and proprioceptive loss (Thoumie and Do 1996; Wang et al. 2022), and inner-ear pathologies (Pyykkö et al. 2022)). For this reason, quantitatively assessing the ability to maintain or reestablish equilibrium is important. To this end, dynamic posturography has been employed to specifically investigate the postural response of subjects to sudden movements of the support base (Baselizadeh et al. 2020; Monaghan and Peterson 2021; Staring et al. 2024) or to perturbations delivered to the body in different ways. For instance, they have been manually imparted to the shoulders of the subjects (Virmani et al. 2023), or obtained by sudden release of cables that support the weight of the body in a forward-leaning position (Thoumie and Do 1996) or by sudden release of a weight pulling the subject forwards or backwards by means of cables attached to a harness worn (Verniba et al. 2023), or by hitting the subject’s trunk by electric actuators (Aprigliano et al. 2019; Zhu et al. 2022; Pacheco Quiñones et al. 2023; Omofuma et al. 2023), by pendulum-like released masses (Duarte et al. 2022; Kaewmanee et al. 2022; Liang et al. 2023) or by hand-held devices (Dvir et al. 2020; Bayón et al. 2021). Regardless of the perturbation type, the postural reaction, a series of coordinated movements of the different body segments, aimed to restore the pre-perturbation state of the body, is commonly detected through center of pressure (CoP) excursion (Baselizadeh et al. 2020; Monaghan and Peterson 2021; Zhu et al. 2022), electromyographic (EMG) signals from postural muscles (Kaewmanee et al. 2022; Liang et al. 2023; Verniba et al. 2023; Staring et al. 2024) or body sway (Jeon et al. 2021; Bayón et al. 2021; Duarte et al. 2022).

A novel automatic device, able to control both force and duration of the perturbation (push) delivered to the upper body (Paterna et al. 2022b; Pacheco Quiñones et al. 2023), was described in recent studies by the authors, along with a postural control index (Δ*CoP*_*n*_), defined as the ratio between the maximum CoP displacement and the perturbation impulse (i.e., the time integral of the force signal). This index showed a good intra-subject repeatability and independence from the perturbation magnitude (Dvir et al. 2020; Paterna et al. 2022a). A dependence of Δ*CoP*_*n*_ on anthropometric characteristics was not evidenced but this result could not be generalized, considering the homogeneous and small-sized sample of 10–14 university students. Thus, this potential influence remained a major concern as it obviously complicates the comparison of postural responses from subjects with different body size and limits the validity of postural indices.

Support to the idea that anthropometric characteristics affect postural control is provided by studies of quiet stance based on a single-link inverted pendulum model, which is still a popular way to characterize the control of human upright posture (Morasso et al. 2019), reporting a specific influence of both height and weight (Hayes 1982; Dames and Richmond 2025). However, the magnitude of the body sway in quiet standing is not necessarily related to the ability to recover balance after postural perturbation (Owings et al. 2000; Mackey and Robinovitch 2005; Tanel et al. 2018). To the best of the authors’ knowledge, only two previous studies have investigated the effects of body size (in obese vs. normal-weight subjects) on the postural response to trunk perturbations (Matrangola and Madigan 2011; Miller et al. 2011). In both studies, a ballistic pendulum was used to apply a pushing force to the participants’ backs. Miller et al. (2011) applied small-amplitude perturbations, while Matrangola and Madigan (2011) applied the maximum perturbation amplitude that could be balanced just by the ankle strategy. In these conditions obese subjects exhibited lower balance recovery limits, both in terms of maximum angular displacement and maximum angular velocity, while the center of mass (CoM) displacement was not significantly different between the two groups.

Based on the above-mentioned few indications from the literature, we hypothesized that the influence of anthropometric variables on the postural response to trunk perturbation, and specifically on the Δ*CoP*_*n*_, could be detected and characterized on a sufficiently large and heterogeneous sample of healthy subjects. The aim of this study was therefore to test this hypothesis, along with the effects of age, and sex. To this goal, an experimental series was conducted based on a previously validated experimental set-up, including an electromechanical system for the delivery of controlled perturbations (Paterna et al. 2022b; Pacheco Quiñones et al. 2023). In addition, the experimental data were tested against the simulations provided by a single-link inverted pendulum model, within the same range of weight and height of the subject sample.

## Methods

### Experimental tests

#### Subjects

A group of 72 healthy adults, 31 female (mean(SD); age: 31(14) years; weight: 61.8(10.3) kg; height: 1.65(0.07) m; foot length: 24.3(0.9) cm) and 41 male (mean(SD); age: 28(12) years; weight: 72.5(11.5) kg; height: 1.76(0.07) m; foot length: 27.2(1.1) cm) was conveniently recruited and tested at a public event focused on general research dissemination targeting non-academic audience. The following exclusion criteria were adopted: recent lower limb injuries or fractures (within 1 year), lower extremity reconstructive surgery in the past, and balance deficits. All subjects provided written informed consent to participate in this study, which was approved by the institutional ethics committee of the University of Torino (Prot. N. 380.583).

#### Task and instrumentation

**Fig. 1:**
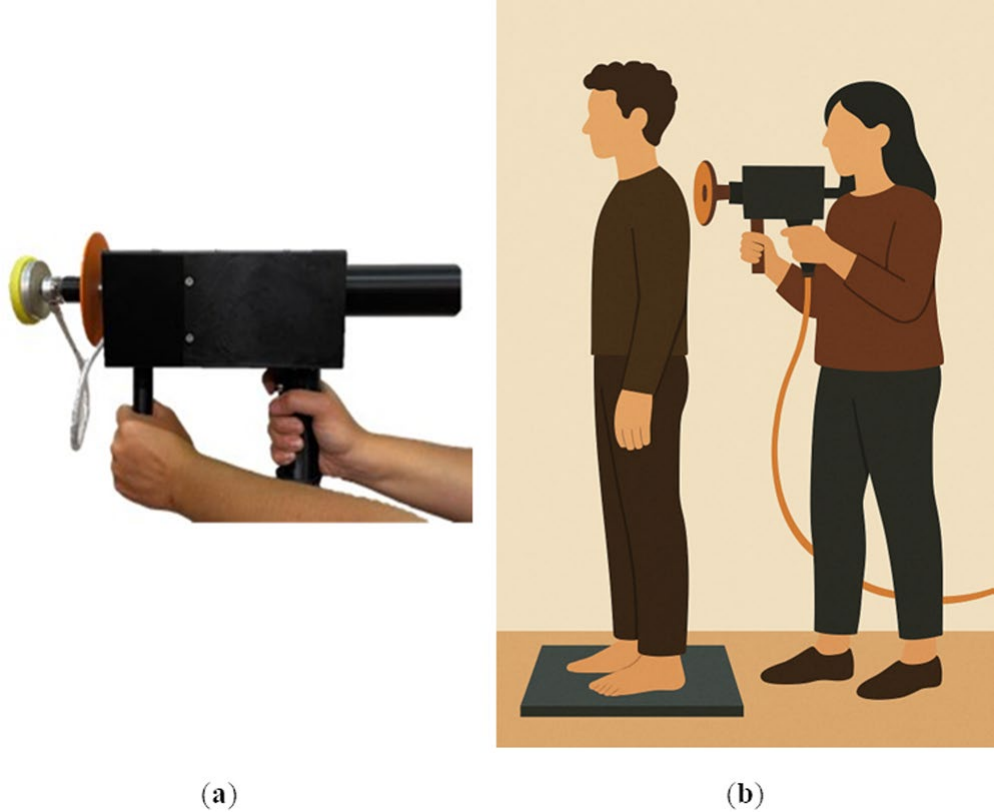
Experimental set-up. a Perturbator device; **b** Example of the experimental task

Subjects were asked to restore equilibrium in response to administered perturbations, consisting of impulsive pushing forces applied to their back while they were standing on a force platform (BMS400600, AMTI, USA). The perturbations were delivered by an electromechanical device, shown in Fig. 1a, and described in detail in previous articles (Paterna et al. 2022b; Pacheco Quiñones et al. 2023). Briefly, the device is held and operated by a skilled operator and delivers well-defined perturbations in both magnitude and duration in a repeatable manner.

After a brief introduction to the purpose of the experiment and the experimental protocol, anthropometric data were collected, including weight, height, and foot length, based on the shoe size. The subjects were then asked to stand on the force platform with their feet at a pelvic distance. The stance width was self-selected by each participant according to the operator’s instructions and qualitatively verified by visual inspection by the operator prior to the start of each trial. Participants stood with their arms at their sides, palms facing inward, and looking forward. They were not instructed to focus on a fixed target, but their orientation (facing a wall of the exhibition stand) and the fact that the area in front of them was not accessible to other people, meant minimal, if any, visual distraction. During the trials, the subjects wore their own clothes and shoes, which allowed for a more relaxed and free participation in the experiment as well as a more natural interaction with the environment. Only in the case of high heels were the subjects asked to remove their shoes.

The operator stood behind the subject and held the perturbator at about 2-cm distance from the subject’s back, targeting the mid-scapular level to deliver a forward perturbation (Fig. 1b). In the beginning, two preliminary perturbations were applied to the subjects to familiarize them with the stimuli. Then 4 consecutive perturbations of 40 N magnitude and 250 ms duration, yielding an impulse of 10 Ns, were delivered. Between one perturbation and the next, a pause of at least 10 s was provided to allow the subject to return to a relaxed stance. A typical testing session lasted less than 5 min.

#### Data processing

**Fig. 2:**
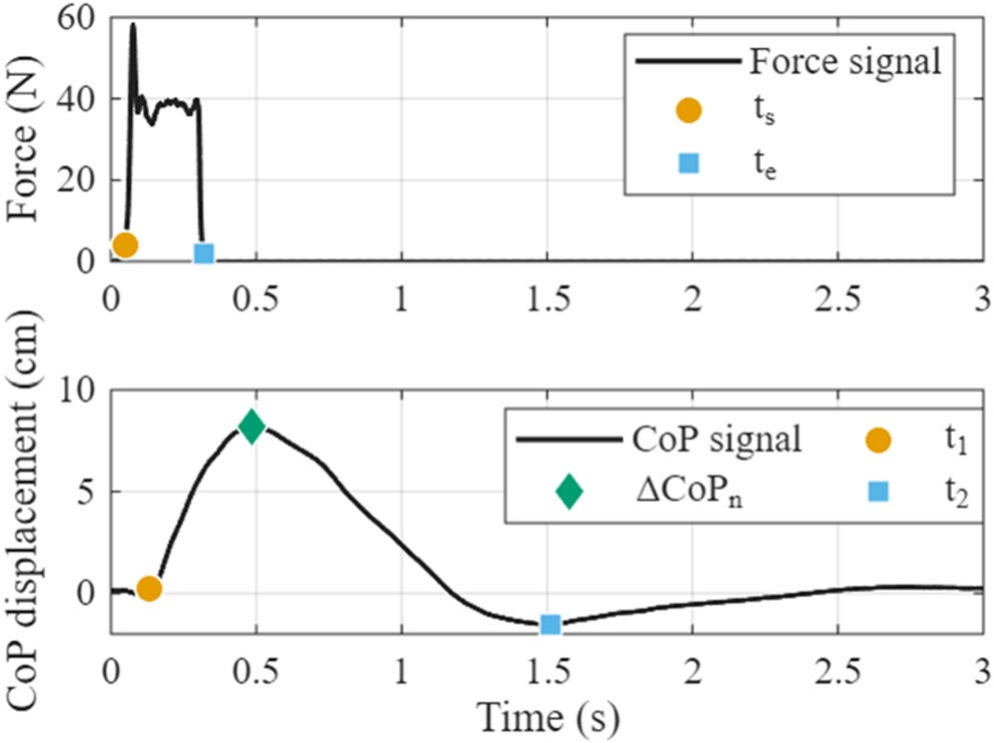
A representative recording of the perturbation (top) and the ensuing postural response (bottom). CoP: center of pressure; t_s_: start of the perturbation; t_e_: end of the perturbation; t_1_: start of the postural response; t_2_: end of the postural response

The perturbation force and the CoP signals were sampled at 1000 Hz and then digitally low-pass filtered with a dual pass 8th order Butterworth filter with cut-off frequencies of 150 Hz and 20 Hz, respectively. A custom MATLAB (release 2023b) routine was developed to extract in post-processing the descriptive parameters of both the perturbation and the CoP displacement. All parameters are described in the following and shown in Fig. 2.

Although the perturbation system was designed to provide stimuli with predefined and constant impulse (*I*), the actual delivered impulse was a posteriori calculated for each perturbation as the force integral throughout the time span defined by the perturbation start (*t*_*s*_) and the perturbation end (*t*_*e*_). Both *t*_*s*_ and *t*_*e*_ were automatically identified as time instants when the force signal crossed the 3.5 N and 1.5 N thresholds, respectively.

As for the postural response, it was characterized in terms of:

- Δ*CoP*_*n*_ (cmN^−1^ s^−1^): the ratio between the maximum CoP displacement (observed within 3 s from *t*_*s*_) and *I*. The maximum CoP displacement was calculated with respect to the average CoP position at rest (i.e., during the 3 s preceding *t*_*s*_).
- Latency (*Lat*, in ms): the time interval from *t*_*s*_ to the start of the CoP response (*t*_1_). The latter was identified based on the maximum of the CoP second derivative.
- Duration of the postural response (*Dur,* in s): the time interval from *t*_1_ to the first local minimum of the CoP displacement after the maximum CoP displacement (*t*_2_).

The correctness of the automatically identified instants of the beginning and end of the perturbation and the postural response was verified by an operator and corrected manually if necessary.

#### Statistical analysis

All statistical procedures were conducted using MATLAB. The predictive relationship between the anthropometric characteristics (age, weight, height, and foot length) and the CoP parameters (Δ*CoP*_*n*_, *Lat*, and *Dur*) was examined using multiple linear regression models. In addition to the regression models, different tests were executed to ensure that model assumptions were met. The Kolmogorov–Smirnov test was used to check the normal distribution of the model residuals; the Breusch-Pagan test was used to assess the residuals’ homoscedasticity; the Durbin-Watson test was used to check the autocorrelation of the residuals. The absence of multicollinearity was tested via the variance inflation factor. Finally, the t-test and the Mann–Whitney test were also used to check the influence of sex on the postural response.

Data in the text are expressed as mean ± standard deviation.

### Single-link inverted pendulum model

A single-link inverted pendulum (SIP) model has been implemented in MATLAB-Simulink (version 2023b) to investigate the theoretical relationship between CoP response and subjects’ anthropometric characteristics.

**Fig. 3:**
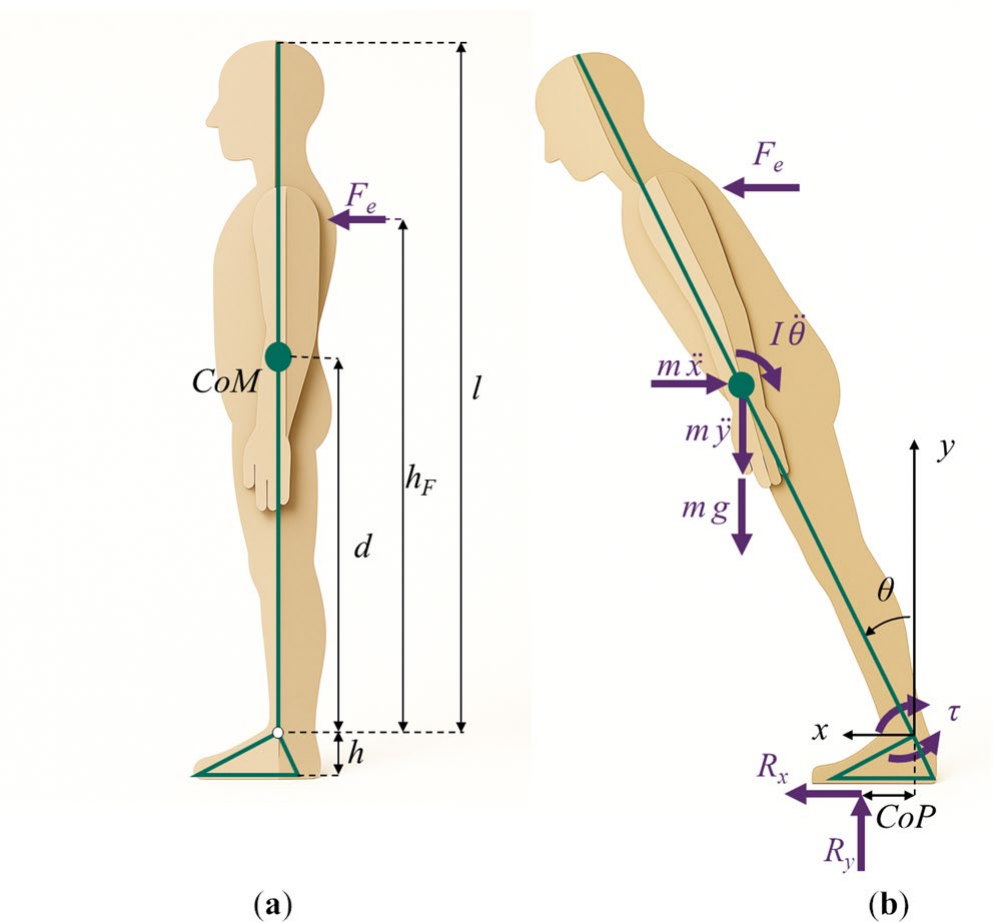
Free-body diagrams for SIP models in resting (**a**) and perturbed (**b**) positions. *θ* is the body oscillation; *l* is the subject height with respect to the ankle joint; *h*_*F*_ is the distance between the ankle joint and the point of application of the perturbation force *F*_*e*_; *d* is the distance between ankle joint and the center of mass (*CoM*); *h* is the height of ankle joint with respect to the base of support; *I* is the rotational inertia of the body about the CoM; *m* is the subject body mass; *ẍ*, *ӱ* and $\ddot{\theta }$ are the horizontal, vertical and angular acceleration of the CoM, respectively; *g* is the gravitational acceleration; *τ* is the correcting torque at the ankle; *CoP* is the center of pressure position; *R*_*x*_ and *R*_*y*_ are the horizontal and vertical components of the ground reaction force

The human body was modeled as a rigid link, perfectly aligned with the vertical direction in resting condition (Fig. 3a), with one rotational degree of freedom around the ankle and the foot firmly in contact with the floor (De Benedictis et al. 2023). During oscillations of the link around the ankle joint, the external load (i.e., the sum of torques due to gravity and to the delivered perturbation) was counteracted by the sum of a passive and an active contribution (Fig. 3b). The passive contribution was mediated by the visco-elastic behavior of the tissue around the ankle joint, as such it was described by stabilizing torque components proportional to the inclination angle (elastic component) and to its first derivative (viscous one) (Engelhart et al. 2015). Conversely, the active contribution, corresponding to the neuromuscular response, was implemented as a delayed, proportional-derivative (PD) action aimed at minimizing the angular position error (van der Kooij et al. 2005; van der Kooij and Peterka 2011; Goodworth and Peterka 2018), that is the value of the current sway angle of the SIP with respect to the vertical. In addition to the traditional proportional and derivative terms, a third term, proportional to the human body’s angular acceleration, was also considered within the active contribution (Pasma et al. 2017).

The ankle torque has been evaluated as expressed by Eqs. 1–3:

1 $$\tau = \tau_{a} + \tau_{p}$$

2 $$\tau_{p} = K\theta_{t} + B\dot{\theta }_{t}$$

3 $$\tau_{a} = K_{p} \theta_{t - T} + K_{d} \dot{\theta }_{t - T} + K_{a} \ddot{\theta }_{t - T}$$

In Eqs. (1–3): *τ*_*a*_ and *τ*_*p*_ are the active and passive component of the ankle torque (τ), respectively; *K* is the passive elastic gain; *B* is the passive viscous gain; *K*_*p*_, is the active control proportional parameter; *K*_*d*_ is the active control derivative parameter; *K*_*a*_ is the active control acceleration-proportional parameter; *θ*, $\dot{\theta }$, $\ddot{\theta }$, are the angular displacement, velocity and acceleration, respectively. Whereas passive torque is synchronous with angular kinematics for each time frame *t* (subscript in Eq. (2)), active torque is subjected to a time delay *T* (subscript in Eq. (3)).

The selection of a SIP model is justified considering that an ankle-based stabilization strategy is generally evoked by low-magnitude perturbations, such as the ones here employed (Horak and Nashner 1986; Shumway-Cook and Woollacott 2017). Moreover, since the stimuli came only from behind in the antero-posterior direction, it seems acceptable to focus on the oscillations resulting in the sagittal plane only. A detailed description of the model can be found in (De Benedictis et al. 2023).

Initially (see Sect. “Modeling individual postural responses”), the model was validated by assessing the accuracy of fitting of the average postural response of individual subjects. To this end, the following procedure was performed for each of the 72 subjects tested:

- The anthropometric parameters of the SIP model were set according to the physical characteristics of the subject considered;
- The passive elastic gain (*K*) was set at 60% of the ankle joint critical stiffness (i.e., the joint stiffness necessary to balance the gravitational torque by considering only the passive contribution available) (Morasso et al. 2019);
- A realistic perturbation signal, calculated as the average of the stimuli provided during the experimental investigation, was fed to the model as input;
- Active control parameters (*K*_*p*_*, K*_*d*_*, K*_*a*_*, T)* and passive viscous gain (*B*) were estimated thanks to a nonlinear least square analysis. The objective function minimized the sum of squared errors between the average experimental CoP response and that predicted by the model;
- The root mean square error (RMSE), the maximum displacement percentage error, and the latency percentage error between the experimental and the modeled response were calculated to estimate the goodness of the fit.

The model was then used to predict the dependence of Δ*CoP*_*n*_ on weight and height, which was achieved by simulating the postural response to the standard stimulus while separately varying weight and height (Sect. “Simulations”). The latter were varied between 60 and 80 kg and 165 cm and 180 cm, respectively, corresponding to the range between the 25th and 75th percentile of the weight and height distribution of the experimental sample. To this aim, the adopted model was the one tuned (according to the procedure described above) on the subject who presented the anthropometric characteristics closest to the median values of the population, here called *median subject*. The predictions of the SIP model were then compared to the results of the linear regression model of experimental data.

## Results

### Experimental results

The impulse of the perturbation was precisely controlled, and its value, averaged among all subjects, was 9.57 ± 0.45 Ns. The perturbations were well tolerated by the subjects, and no stepping reaction was observed.

**Fig. 4:**
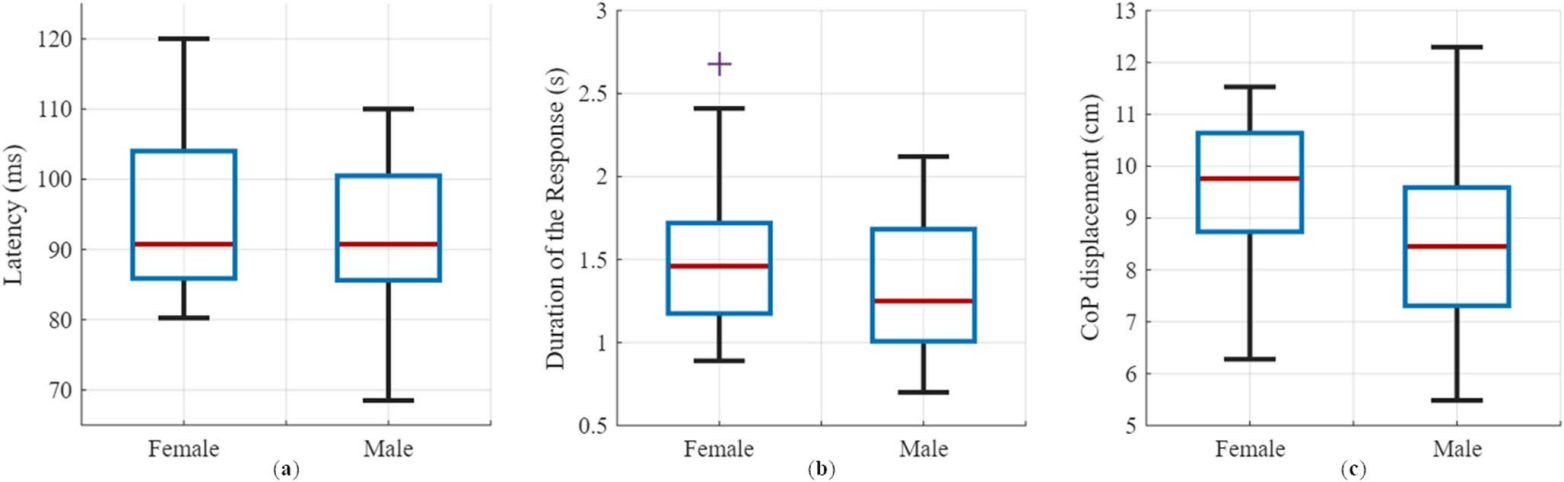
Distribution of the latency (**a**), duration (**b**) and maximum displacement (**c**) of the CoP postural response with respect to sex. (n_Female_ = 31; n_Male_ = 41). On each box: the central red line indicates the median; the bottom and top edge of the blue box indicate the 25th and 75th percentiles; the whiskers represent the extreme data points; the ‘ + ’ marker represent the outlier

The CoP response was delayed with respect to the start of perturbation (Fig. 2). On average, the response latency was 96.15 ± 12.16 ms for the female group, and 91.52 ± 9.75 ms for the male group. In addition, the whole response lasted 1.52 ± 0.47 s, and 1.32 ± 0.37 s, and the maximum CoP displacement was 9.55 ± 1.37 cm and 8.55 ± 1.47 cm for females and males, respectively. The distribution of the CoP parameters is shown in Fig. 4. Differences between males and females were not statistically significant.

**Fig. 5:**
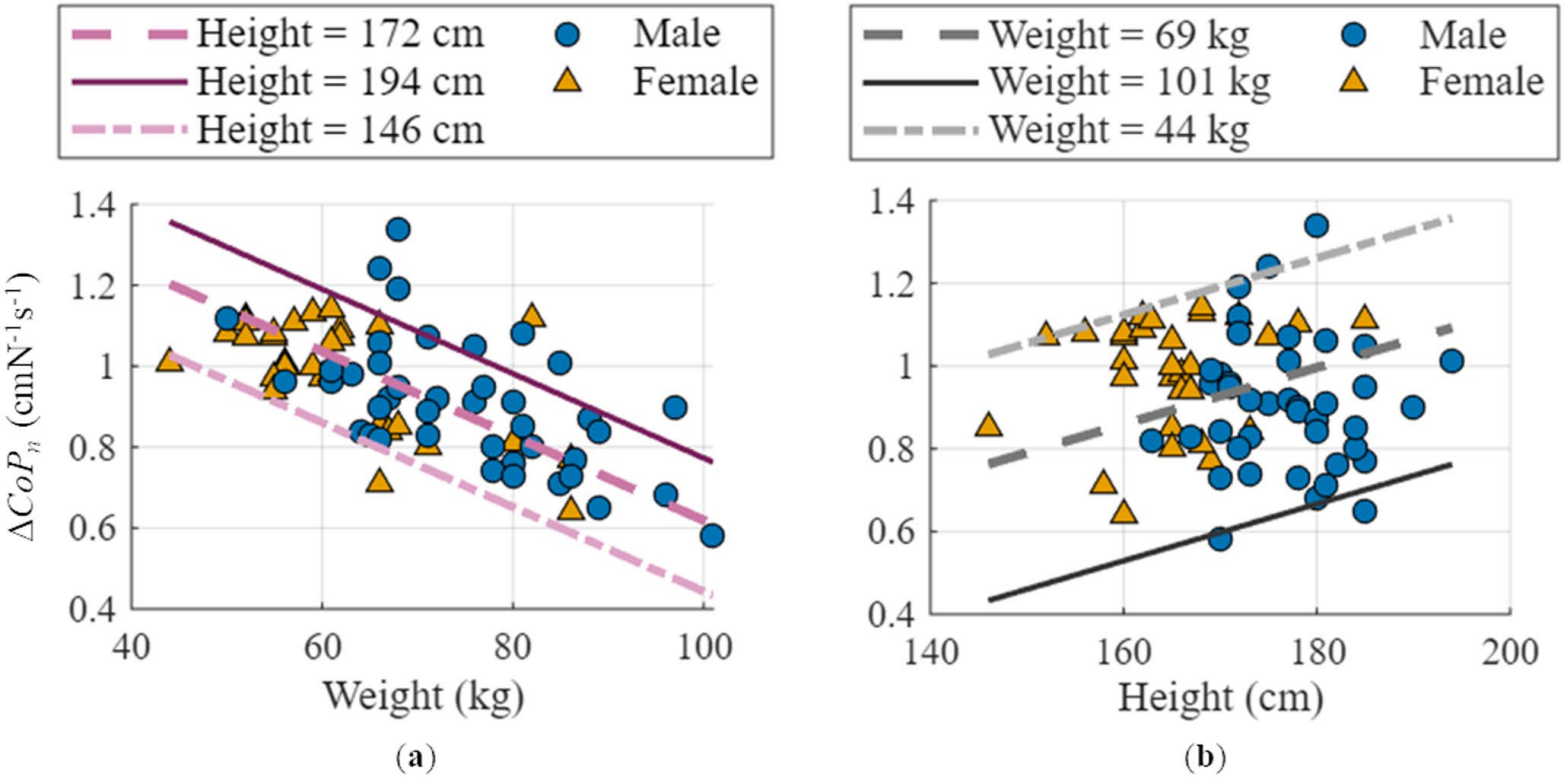
Dependence of Δ*CoP*_*n*_ on weight (**a**) and height (**b**) of the subjects. Purple lines represent linear regression lines calculated for different subject height values. Grey lines represent linear regression lines calculated for different subject weight values

The first linear regression analysis aimed to assess the impact of sex, age, and anthropometric characteristics (weight, height, and foot length) on Δ*CoP*_*n*_. A stepwise regression revealed a statistically significant model (F_(2,69)_ = 33.6, p-value = 6.6e − 11), between Δ*CoP*_*n*_, weight, and height (Fig. 5). The adjusted R^2^ of the model (Eq. 4) is 0.48, indicating that the model accounts for approximately 48% of the Δ*CoP*_*n*_ variability.

4 $$\Delta CoP_{n} = 0.48415 - 0.01043{*}W + 0.0068648{*}H$$

In Eq. 4, *W* is the subject weight (in kg) while *H* is the subject height (in cm).

For each additional kg of body weight, there is an average decrease of 0.01 cmN^−1^ s^−1^in Δ*CoP*_*n*_, with a 95% confidence interval of [− 0.0117; − 0.0091]. This negative relationship between weight and Δ*CoP*_*n*_ is statistically significant (t_(69)_ =  − 8.0976, *p*-value = 1.3e − 11). On the other hand, an increase of 1 cm in body height contributes to Δ*CoP*_*n*_ increase of about 0.007 cmN^−1^ s^−1^, with a 95% confidence interval of [0.0051; 0.0086]. This positive relationship between height and Δ*CoP*_*n*_ is statistically significant (t_(69)_ = 3.8489, *p*-value = 2.62e − 04). The standardized linear regression coefficients are − 0.2945 for weight and 0.1400 for height, respectively. As a result, weight has a greater influence on Δ*CoP*_*n*_. The residuals of the regression analysis were examined to ensure that the model assumptions were met. The residuals were normally distributed (Kolmogorov–Smirnov, *p*-value = 0.48), homoscedastic (Breush-Pagan, *p*-value = 0.63), and were not autocorrelated (Durbin-Watson, *p*-value = 0.73). Furthermore, the visual analysis of the residuals’ scatter plot did not highlight a discernible pattern. Finally, the variance inflation factor is 1.56, excluding a linear relationship between subjects’ weight and height.

On the other hand, no significant correlation between the anthropometric characteristics and the other two CoP parameters, *Lat* and *Dur*, was detected by the matrix correlation plots. In addition, sex affected neither *Lat* (Mann–Whitney test, *p* = 0.25) nor *Dur* (t-test, *p* = 0.1), as can also be seen from the boxplots of Fig. 4.

### Modeling individual postural responses

**Fig. 6:**
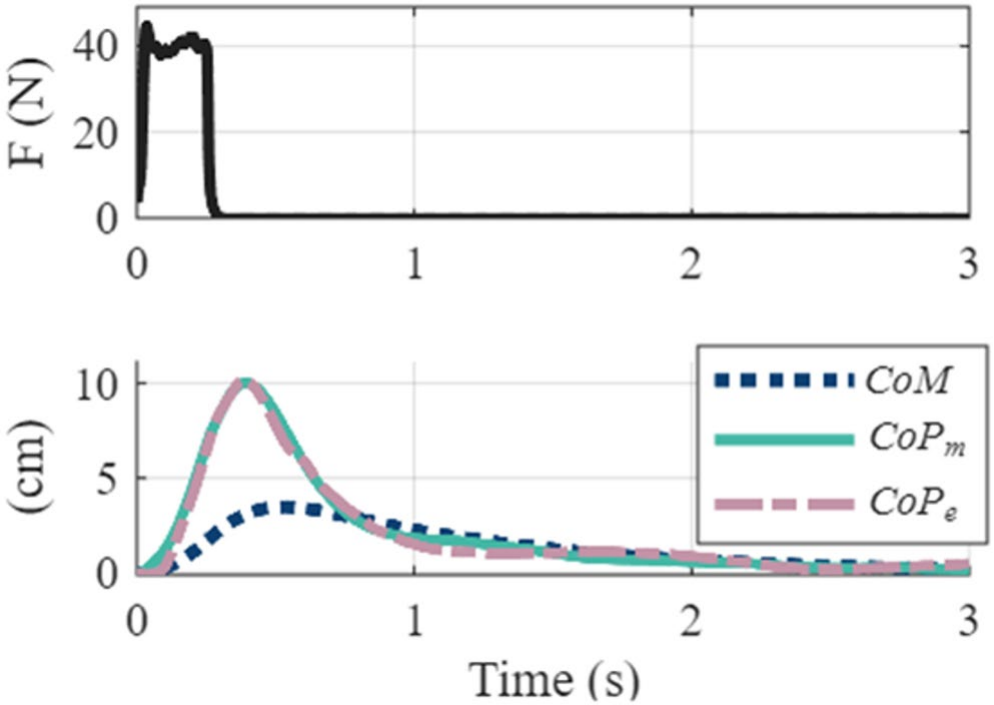
Perturbation (top) and CoP fit (bottom) for a representative subject being modeled as SIP. *CoP*_*e*_: average CoP response collected from the experimental recordings from the subject; *CoP*_*m*_: simulated CoP response provided by the model; *CoM*: simulated CoM response

Figure 6 depicts the results provided by the SIP model tuned to fit the postural response (CoP_e_, average of 5 responses) of a representative subject. Along with the fitting curve (*CoP*_*m,*_ Fig. 6 turquoise line), the model also simulates the time course of other biomechanical variables such as the CoM, also displayed in Fig. 6 (blue line).

**Fig. 7:**
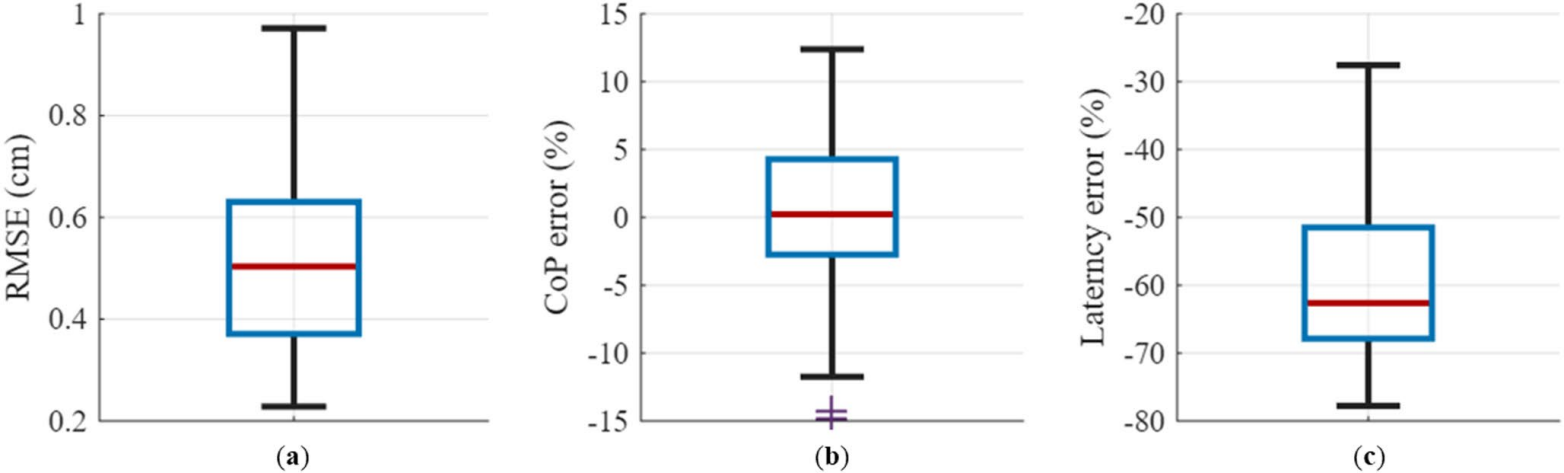
Distribution of the CoP displacement root mean square error (RMSE) (**a**), the maximum CoP displacement percentage error (**b**) and the latency percentage error (**c**). (n = 72). On each box: the central red line indicates the median; the bottom and top edge of the blue box indicate the 25th and 75th percentiles; the whiskers represent the extreme data points; the ‘ + ’ markers represent the outliers

As graphically shown in Fig. 7, the quality of fitting was generally good as indicated by the RMSE, being less than 1 cm, and the absolute value of the maximum CoP displacement percentage error, on average 4.83%. A large error was instead observed in the assessment of the latency of the response (on average –59%), which is however caused by a small discrepancy between the two curves immediately after the perturbation, namely, an early rise of the simulated CoP response, compared to the experimental.

### Simulations

As explained in the Methods, the SIP model was first loaded with the anthropometric data of the *median subject*, identified as the subject more closely approaching the median values of the experimental sample (66.5 kg, 171.5 cm), who happened to be a male with mass of 67 kg, and height of 173 cm. The model parameters (*B, K*_*p*_*, K*_*d*_*, K*_*a*_*, T*) were then estimated by fitting his postural response (see all model parameters in Table 1). The fully configured SIP model was then used to predict the Δ*CoP*_*n*_ dependence on weight and height.

**Table 1:** Optimized model parameters. *B* is the passive viscous gain; *K*_*p*_ and *K*_*d*_ are the control proportional and derivative terms; *K*_*a*_ is the acceleration proportional parameter; and *T* is the control time delay

Parameter	Value
*B*	137.8534 Nms/rad
*K*_*p*_	416.4054 Nm/rad
*K*_*d*_	141.0696 Nms/rad
*K*_*a*_	0 Nms^2^/rad
*T*	207 ms

**Fig. 8:**
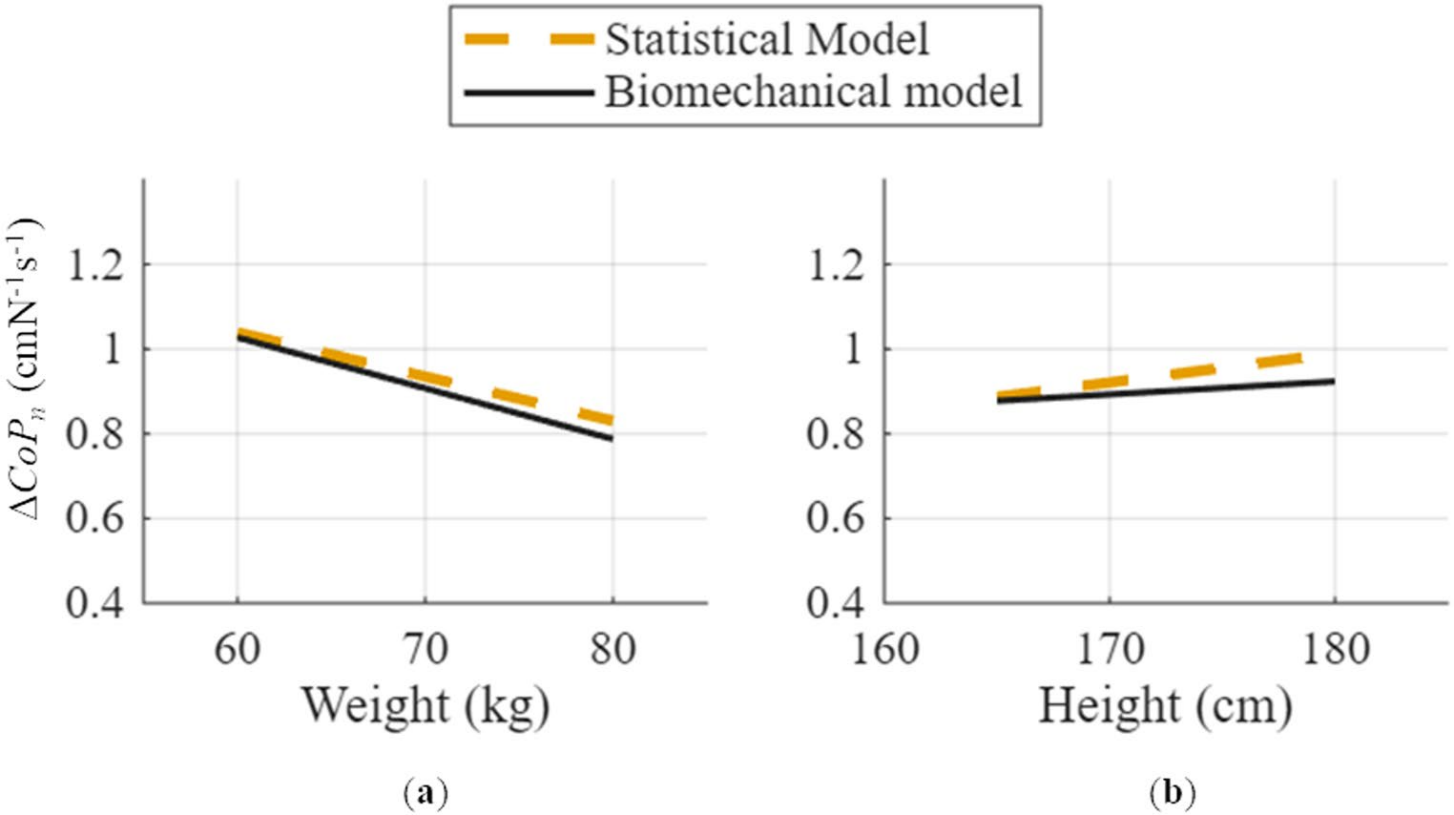
Comparison between the statistical and biomechanical models. **a ***CoP* trend as the subject weight changes (height = 172 cm); **b ***CoP* trend as the subject height changes (weight = 70 kg)

In accordance with the experimental results, the simulation confirms a decrease in Δ*CoP*_*n*_ as weight increases (solid line in Fig. 8a) and an increase with increasing height (solid line in Fig. 8b). Notably, the simulation closely matches both absolute values and slopes of the experimental data (orange dashed lines in Fig. 8), particularly in the Δ*CoP*_*n*_ − weight relation.

**Fig. 9:**
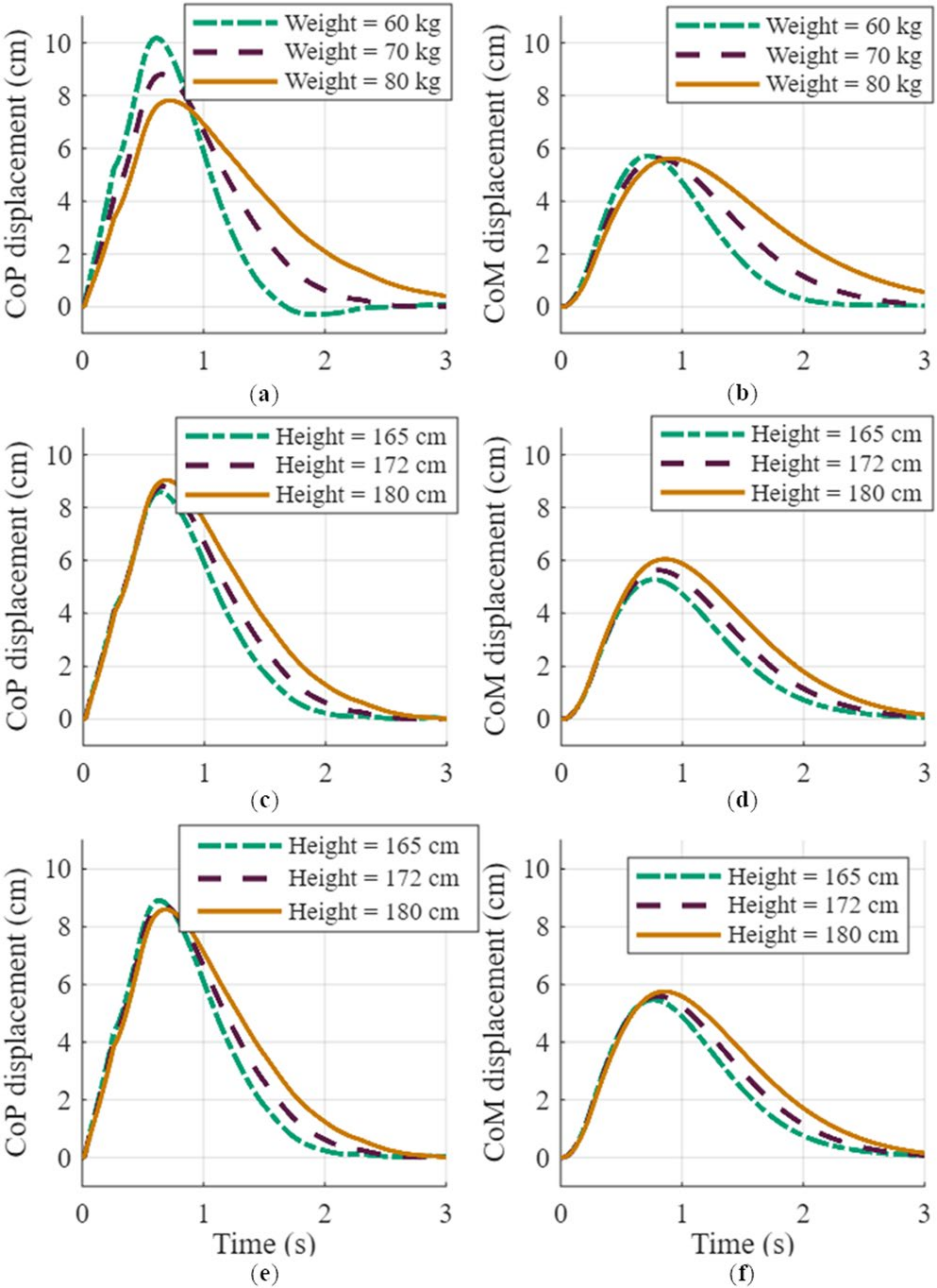
*CoP*_*m*_ and *CoM* dependence from weight (**a**, **b**) and height (**c**–**f**) of the subject (body height set at 172 cm). In c and d the distance between the perturbation application point and the ankle joint was maintained proportional to the subject’s height (body weight set at 70 kg); while in e and f the perturbation was always applied at a distance of 126 cm from the ankle (body weight set at 70 kg)

This effect can also be observed from the results of single simulations: the time courses of *CoP*_*m*_ and *CoM* displacement for three different body mass values are depicted in Fig. 9a, b. As the body mass increases, the *CoM* maximum displacement remains almost constant, while the maximum displacement of the *CoP*_*m*_ steadily decreases. If body height is increased (while at the same time increasing the height of perturbation delivery, as was done experimentally by application of the perturbation always at interscapular level), the maximum *CoP*_*m*_ increases by a limited amount, while there is a noticeable increase in the maximum *CoM* (Fig. 9c, d). However, if the height of perturbation delivery is maintained unchanged, the magnitude of CoP and CoM responses remains mostly unaffected by changes in body height (Fig. 9e, f).

## Discussion

This study aimed to investigate the effect of anthropometry, age and sex on the postural response to external impulsive perturbations applied to the trunk. Knowing the relationship between postural response and anthropometry is fundamental to correctly interpret the postural response and adapt the posturographic test to the characteristics of the patients.

A custom-made electromechanical device that delivers well-defined and repeatable mechanical postural disturbance was employed to reduce the inter-subject variability of the stimuli. This allowed the test to be reduced to only 4 perturbations per subject. The residual variability in the delivered perturbation, possibly introduced by the operator handling the perturbation device was confirmed to be low enough (< 5%) for the correct realization of the trials.

The subject’s physical characteristics did not affect the duration and latency of the postural response, which is not surprising for the latter parameter in particular, being related to the timing of postural reflexes. On the other hand, the experimental and simulation tests showed that Δ*CoP*_*n*_ decreased with increasing subject weight and decreasing height. These two outcomes will be separately discussed.

### Influence of body weight

The influence of body weight on postural control has been addressed in several studies, but the results are not homogeneous. De Oliveira (2017) observed that, during eyes-open static posturography tests, the area of the statokinesiogram (calculated as the area of the confidence ellipse) increases as the subjects’ weight increases. Hue et al. (2007) and Menegoni et al. (2009) reported that the mean CoP speed increases with body weight during quiet stance, regardless of whether subjects keep their eyes open or closed. Greve et al. (2013) and De Maio et al. (2021) asked the subjects to stand on a sensorized wobble board and measured, respectively, the variance of the tilt angle and how long the subjects maintained a tilt angle close to zero. Their results showed that as body weight increased, tilt angle variance also increased while the wobble board was horizontal for shorter durations. Based on the assumption that smaller and slower movements of the CoP indicate greater postural stability, all of these studies concluded that higher body weight was responsible for worsened performance. In contrast, Cieślińska-Świder and Błaszczyk (2019) and Błaszczyk and Cieślińska-Świder (2019) observed a decrease in mediolateral CoP speed and mean CoP speed, respectively, in obese subjects with respect to normal-weight individuals. They hypothesized that the increased support base and higher body inertia associated with greater body mass enhanced postural stability under quiet conditions.

Besides these contrasting results, it should also be considered that the mechanisms underlying CoP changes in static conditions may not adequately predict postural control in dynamic conditions. Few studies have compared responses of lean and obese subjects to dynamic postural challenges, again with contrasting results. Berrigan et al (2006) reported increased CoP displacement and speed in obese subjects performing goal-oriented movements, thus suggesting poorer control of balance with respect to non-obese subjects. However, with self-induced-perturbations, the magnitude of the perturbation is not known and is not the same for all subjects but depends on their characteristics. Opposite conclusions were reported by Miller et al. (2011), who observed that in response to low-magnitude impulsive perturbations (≤ 8 Ns), the mean CoM velocity was lower in obese individuals compared to normal-weight counterparts. Interestingly, they also showed that when the perturbation magnitude was normalized to body weight, no significant differences in CoM kinematics could be observed between the two groups. These findings suggest that the influence of increased weight and inertia may depend on the type of perturbation as was further demonstrated in a study which looked into different perturbation types (2011). The present results support this view. All participants of the current study received a carefully controlled impulsive perturbation of 10 Ns, so, all of them were subjected to approximately the same change in momentum but the imparted CoM velocity would decrease with increasing body weight and inertia, thus requiring a lower CoP response to maintain the balance, as clearly indicated by the experimental results and model simulations. Consequently, perturbations are more challenging for lighter subjects. In addition, the simulation results show that, by increasing the body weight, the CoM velocity is lower, in agreement with Miller et al (2011), and the CoM takes longer to return to its equilibrium position (Fig. 8b), likely due to the increased inertia. Based on the above considerations, we suggest that in response to small forward perturbations, the reduction in Δ*CoP*_*n*_ with increasing body weight does not reflect an improvement in postural performance but simply results from biomechanical factors. Importantly, thanks to the large sample size, the influence of body weight on the postural response has been modeled by a linear relation and the reliability of this approximation was confirmed by a simple SIP model. This model provides, for the first time, a reasonable explanation for a relevant confounding factor in postural control assessment.

### Influence of body height

One of the mechanical principles of stability is that it is inversely related to the height of the CoM from the support base (Hayes 1982). There is a consensus that the postural performance deteriorates with body height (Greve et al. 2013; De Oliveira 2017). In previous studies, contrasting indications of non-significant or negative linear correlation between Δ*CoP*_*n*_ and height in response to forward perturbations were reported (Dvir et al. 2020; Paterna et al. 2022a). However, the sample was limited in size and characterized by a narrow range of body mass index (BMI: from 18.4 to 23.9 kg/m^2^). Therefore, the reduction in Δ*CoP*_*n*_ with increasing height was likely confounded by body mass, as taller individuals in the sample were also heavier. The present results, from a much larger and diverse subject population, bring support to the general rule, evidencing a weak but significant positive relation between height and Δ*CoP*_*n*_. Furthermore, the relation was confirmed by the SIP model which also evidenced similar dependence of CoM on height. Importantly, the SIP model allowed the separation of contributions derived from the weight and the height by simulating postural responses to selective changes in one or the other variable. In addition, it revealed that the influence of the subject’s height on the postural response was secondary to the corresponding change in the height of the point of application of the perturbation. In fact, by applying the perturbation at the interscapular level, its lever arm with respect to the ankle fulcrum increased with the subject’s height, thus amplifying the imparted angular momentum and the ensuing changes in CoM and CoP. These effects were avoided when the application point of the perturbation was maintained at a fixed height from the ankle, rather than at the interscapular point (Fig. 9e, f). The simulation was crucial to clarify this issue.

The present results suggest that controlling for the impulse of the perturbation rather than force may not be sufficient to standardize the perturbation and that a constant angular momentum could be a better choice. This could possibly be achieved by scaling the impulse according to the subject height (the taller the subject, the smaller the impulse) or by correcting a posteriori the postural response possibly permitting a more meaningful comparison of balance control between subjects.

### Agreement between the statistical and the SIP models

The good agreement resulting between the statistical linear model of experimental data and the predictions yielded by the SIP model, previously tuned on the CoP response of the *median subject*, represents an important cross-validation of the two approaches suggesting that 1) the data set was sufficiently large and diverse to produce reliable statistical estimates; 2) the simplified SIP model with a single degree of freedom was adequate to describe the postural reactions to weak forward perturbations, exclusively based on the ankle strategy. There are, however, a few issues worth to be discussed.

In contrast to the experimental data, where the CoP response presents a latency in the order of 90–100 ms with respect to the perturbation, the simulated CoP response instantaneously rises, virtually with no latency, at the very onset of the perturbation. This behavior is due to the large contribution of the passive ankle torque, particularly its viscous component. As a result, the current SIP model does not allow for a meaningful analysis of the CoP latency. Future studies should include more realistic simulations by considering models with more degrees of freedom (multiple-link inverted pendulum models (Goodworth and Peterka 2018)) and with more sophisticated control laws. Moreover, the integration in the optimization procedure of additional signals from the experimental field, such as body kinematics and the tangential forces exchanged between feet and platform, may serve to achieve a more accurate fit of the postural response.

The slight differences between the statistical (Fig. 8, dashed orange lines) and the biomechanical model regression lines (Fig. 8, solid lines) may be explained by the simplified model assumptions. Although previous studies (Simoneau and Teasdale 2015; Bollinger 2017) showed that obese individuals have greater ankle stiffness and damping than normal-weight subjects, only the passive elastic gain, *K*, was adjusted in simulations in which weight and height are varied (others ankle torque model parameters are kept constant). Moreover, in the SIP model, the CoM is assumed to lie on the human body’s longitudinal axis and at a distance from the ankles, which depends only on the subject’s height. However, especially in overweight subjects, the position of the CoM is influenced by the distribution of adipose tissue. For instance, abdominal fat accumulation tends to move the CoM anteriorly, whereas adipose tissue concentrated in the thighs may result in a downward CoM displacement (Ahn et al. 2024). These considerations suggest that there might indeed be a systematic influence of anthropometric characteristics on the parameters of the biomechanical model, which may account for the small differences of Fig. 8.

### Limitations

A possible limitation of the study is that the data were not collected under the carefully controlled conditions of a research laboratory, but at a stand of a public event where noise from adjacent stands, comments and questions from other visitors, and distractions could not be fully prevented. In addition, the volunteers wore their own clothes and shoes. We cannot exclude that these disturbing factors may have introduced some variability in the collected data. On the other hand, this is the type of environment we normally live in and where unexpected perturbations may occur and put our balance at risk. On this basis, results obtained in field experiments may be particularly meaningful. The good match obtained between experimental and simulated data suggests that the data collected were indeed reliable and adequate to extract solid conclusions.

A second limitation of this study is that foot length was estimated from shoe size rather than measured directly. This indirect approach may have led to imprecise estimates of actual foot length. Therefore, future studies should re-examine the potential dependence of CoP parameters on foot length by directly measuring this anthropometric variable, in order to confirm the absence of relationship between *ΔCoP*_*n*_ and foot length.

Finally, the adopted SIP model, as mentioned above, was not adequate to fully describe the features of the postural response, in particular its latency. This simple model was however adequate to outline the influence of weight and height of the subject and of the height of the application point.

## Conclusion

Experimental results and model simulations consistently highlighted a linear relationship between the magnitude of the CoP response to unexpected postural perturbations imparted to the trunk, and the weight and height of the subjects. On the contrary, latency and the duration of the CoP response were not affected by the physical characteristics of the subject. These results have implications for balance assessment: further normalization of Δ*CoP*_*n*_ according to the subject’s weight and height may eliminate or limit the influence of these confounding factors, improve the objectivity of the measurement and favor inter-subject comparison of the postural control capacity.

## Data Availability

The data that support the findings of this study are available from the authors on reasonable request.

## Abbreviations

CoP: Center of Pressure
*CoP*_*m*_: Center of Pressure (simulated by the model)
CoM: Center of Mass
Δ*CoP*_*n*_: Ratio between maximum CoP displacement and perturbation impulse
*Dur*: Duration (length of postural response)
*Lat*: Latency (onset time of postural response)
SIP: Single-link Inverted Pendulum
